# Revisiting the Phylogenetic History of Helminths Through Genomics, the Case of the New *Echinococcus oligarthrus* Genome

**DOI:** 10.3389/fgene.2019.00708

**Published:** 2019-08-07

**Authors:** Lucas L. Maldonado, Juan Pablo Arrabal, Mara Cecilia Rosenzvit, Guilherme Corrêa De Oliveira, Laura Kamenetzky

**Affiliations:** ^1^IMPaM, CONICET, Facultad de Medicina, Universidad de Buenos Aires, Buenos Aires, Argentina; ^2^INMet, Instituto Nacional de Medicina Tropical, Puerto Iguazú, Argentina; ^3^Instituto Tecnológico Vale, Belém, Brazil

**Keywords:** *Echinococcus oligarthrus*, genome, phylogeny, single nucleotide polymorphism, chromosomes, parasites

## Abstract

The first parasitic helminth genome sequence was published in 2007; since then, only ∼200 genomes have become available, most of them being draft assemblies. Nevertheless, despite the medical and economical global impact of helminthic infections, parasite genomes in public databases are underrepresented. Recently, through an integrative approach involving morphological, genetic, and ecological aspects, we have demonstrated that the complete life cycle of *Echinococcus oligarthrus* (Cestoda: Taeniidae) is present in South America. The neotropical *E. oligarthrus* parasite is capable of developing in any felid species and producing human infections. Neotropical echinococcosis is poorly understood yet and requires a complex medical examination to provide the appropriate intervention. Only a few cases of echinococcosis have been unequivocally identified and reported as a consequence of *E. oligarthrus* infections. Regarding phylogenetics, the analyses of mitogenomes and nuclear datasets have resulted in discordant topologies, and there is no unequivocal taxonomic classification of *Echinococcus* species so far. In this work, we sequenced and assembled the genome of *E. oligarthrus* that was isolated from agoutis (*Dasyprocta azarae*) naturally infected and performed the first comparative genomic study of a neotropical *Echinococcus* species. The *E. oligarthrus* genome assembly consisted of 86.22 Mb which showed ∼90% identity and 76.3% coverage with *Echinococcus multilocularis* and contained the 85.0% of the total expected genes. Genetic variants analysis of whole genome revealed a higher rate of intraspecific genetic variability (23,301 SNPs; 0.22 SNPs/kb) rather than for the genomes of *E. multilocularis* and *Echinococcus canadensis* G7 but lower with respect to *Echinococcus granulosus* G1. Comparative genomics against *E. multilocularis*, *E. granulosus* G1, and *E. canadensis* G7 revealed 38,762, 125,147, and 170,049 homozygous polymorphic sites, respectively, indicating a higher genetic distance between *E. oligarthrus* and *E. granulosus sensu lato* species. The SNP distribution in chromosomes revealed a higher SNP density in the longest chromosomes. Phylogenetic analysis using whole-genome SNPs demonstrated that *E. oligarthrus* is one of the basal species of the genus *Echinococcus* and is phylogenetically closer to *E. multilocularis*. This work sheds light on the *Echinococcus* phylogeny and settles the basis to study sylvatic *Echinococcus* species and their developmental evolutionary features.

## Highlights

- Whole-genome SNP analysis showed high divergence among *Echinococcus sensu lato*.
- *Echinococcus* chromosomes have differential distribution of single-nucleotide polymorphisms.
- Sylvatic *Echinococcus* species are phylogenetically closer to each other than *Echinococcus granulosus sensu lato* species.
- Phylogenetic analysis demonstrated that *Echinococcus oligarthrus* is one of the basal species.

## Introduction

Helminth parasites are a highly diverse group that involves many parasites of biomedical, veterinary, and economic importance including roundworms (nematodes) and flatworms (Platyhelminthes: trematodes and cestodes). Taxonomic assignment of the parasitic helminth taxa is a particularly arduous task. Indeed, parasites are typically difficult to culture and analyze independently of their hosts and the parasite body fossil samples are scarce due to their small size, lack of hard parts, and their lifestyle within the host. In addition, molecular analyses do not often include all the species or include only partial sequences (De Baets et al., 2015). Among Platyhelminthes, the classification and nomenclature within the genus *Echinococcus* is a controversial topic (McManus, 2013; Lymbery et al., 2015). The strobilar stage of these parasites occurs in the small intestine of a definitive carnivore host; the metacestodes develop in the organs of an herbivorous intermediate host that is the prey of the final hosts. In the last decades, several subspecies of *Echinococcus granulosus* were proposed based mainly on the intermediate host specificity (Verster, 1969). However, most of the early proposed subspecific taxa were relegated to synonymy under the name *E. granulosus* due to their sympatric distributions and because they are indistinguishable at the morphological level (Rausch et al., 1967). Consequently, based on the production of a distinctive form of echinococcosis in humans, only four species had been retained: *E. granulosus sensu lato*, which causes cystic echinococcosis; *Echinococcus oligarthrus*, which causes unicystic echinococcosis; *Echinococcus vogeli*, which causes polycystic echinococcosis; and *Echinococcus multilocularis*, which causes alveolar echinococcosis. The pathogenicity degree of the echinococcosis infections depends on the characteristics of the metacestode development, which is different in each one of the four species mentioned before (Lymbery, 2017). Nowadays, genetic and genomic mitochondrial analysis allows the revision of the phylogeny of the *Echinococcus* genus determining the species rank for nine taxa: *E. granulosus sensu stricto*, *Echinococcus canadensis*, *Echinococcus ortleppi*, *Echinococcus equinus*, *Echinococcus felidis*, *E. oligarthrus*, *E. vogeli*, *E. multilocularis*, and *Echinococcus shiquicus* (Xiao et al., 2006; Nakao et al., 2007; Hüttner et al., 2008; Nakao et al., 2013a; Kinkar et al., 2017). Nuclear DNA has also been used to reconstruct the phylogeny of taeniid parasites, which differs from the phylogeny obtained with mitochondrial data (Saarma et al., 2009; Knapp et al., 2011). Even though there are certain common features in the taxonomy regardless of the origin of the molecular data. *E. felidis* and *E. granulosus s. s.* are sister species, and *E. ortleppi* is closely related to the different genotypes of *E. canadensis*. However, the position of the neotropical species, *E. vogeli* and *E. oligarthrus* (basal or non-basal), and whether *E. multilocularis* and *E. shiquicus* are sister species remain unknown (Lymbery, 2017). Several authors agree that further analyses using more nuclear DNA sequences are required in order to completely resolve the relationships among putative species within the genus (Saarma et al., 2009; Nakao et al., 2013a; Lymbery, 2017). Particularly, neotropical species have been the least studied and only a few cases have been reported and published so far (D’Alessandro and Rausch, 2008; Soares et al., 2013; Arrabal et al., 2017). Hence, a better sampling and understanding of the neotropical species will help to resolve the *Echinococcus* phylogeny. In 2013, with the publication of the first tapeworm genomes that included *E. multilocularis* and *E. granulosus* G1 species (Tsai et al., 2013; Zheng et al., 2013), the “Tapeworm genome era” began. In 2017, we sequenced and assembled the *E. canadensis* G7 genome and performed several comparative genomic analyses confirming the species status of the taxa (Maldonado et al., 2017). In this work, with the aim of obtaining a deeper description of the *Echinococcus* phylogeny, we sequenced the complete *E. oligarthrus* genome and performed whole-genome variant analysis among all the *Echinococcus* species that are currently available. The results presented here demonstrate the basal origin of the neotropical *Echinococcus* species and propose that the use of complete genome data is crucial for the unequivocal helminth phylogenetic studies.

## Materials and Methods

### Sample Collection, DNA Extraction, and Next-Generation Sequencing

#### Parasites Material

*E. oligarthrus* cysts were collected from Iguazú National Park, in the North of Misiones province, Argentina. Cysts were obtained from the livers of naturally infected agoutis (*Dasyprocta azarae*). The animals involved in this study were not subjected to any experimental procedure. All the samples used in this study were collected post-mortem from road-killed animals. For genome sequencing purposes, protoscoleces were aseptically removed from the cysts and extensively washed in phosphate buffer saline and visualized under an optical microscope. The species and genotype were determined by sequencing a fragment of the mitochondrial cytochrome C oxidase subunit 1 (COX1) (Arrabal et al., 2017).

#### DNA Isolation, Library Construction, and DNA Sequencing

The isolation of high-quality genomic DNA was performed by the phenol/chloroform method as previously described (Maldonado et al., 2017). Briefly, the samples were quantified using a Qubit Fluorometer (Invitrogen), the quality was evaluated by the OD rate 260/280 and 260/230 using a NanoDrop (ThermoFisher Scientific). MiSeq Illumina libraries were prepared as follows. For each library preparation, 50 ng of DNA was subjected to a random tagmentation reaction, and DNA was simultaneously fragmented and linked to specific adapters using the Nextera^®^ XT DNA Sample Preparation Kit, according to the manufacturer’s instructions. Two libraries of 530-bp fragment size were obtained and subjected to 500 sequencing cycles (2 × 250 bp) using the MiSeq v2 Reagent Kit. The quality of the Illumina reads was evaluated with FastQC v0.10.1, and the reads were trimmed and end-clipped to a Phred score of 33 using Trimmomatic (Bolger et al., 2014).

### *De Novo* Assembly of *E. oligarthrus* NGS Reads

The genome of *E. oligarthrus* was assembled from a combination of two paired-end libraries sequenced in the Illumina MiSeq platform. The genome assembly involved several steps. First, a preliminary *de novo* assembly using SPAdes 3.6 (Bankevich et al., 2012; Safonova et al., 2014) was performed. The assembly sequences were screened against the NCBI nt database, using Nucleotide–Nucleotide BLAST v2.6.0+ (available at ftp://ftp.ncbi.nlm.nih.gov/blast/db/FASTA/nt.gz) in megablast mode, with an e-value cutoff of 1e−25 and a culling limit of 2 and using DIAMOND tblastx against SwissProt (Buchfink et al., 2015). Raw, paired-end Illumina reads were mapped against the assembly using Bowtie2 (Langmead and Salzberg, 2012). The output was converted to a BAM file using Samtools (Li and Durbin, 2009). Blobtools v1.1 (Laetsch and Blaxter, 2017) was used to create taxon-annotated GC-coverage plots for *E. oligarthrus* genome assembly and to identify the target sequences and target reads using as input the Nucleotide–Nucleotide and DIAMOND BLAST (Buchfink et al., 2015) and the raw read mapping results. Sequences that did not match the Platyhelminthes taxon as a top BLAST hit at the phylum level were filtered out. After removing contaminants, sequences that did match as a top BLAST hit at the phylum level and whose base coverage was >4× were used to recover the target reads. Before the assembly, we used Jellyfish to create a k-mer histogram and the convergence was analyzed with genomescope in order to evaluate whether after cleaning with blobtools the remaining reads could be used to obtain an acceptable assembly. The target reads extracted from the bam files were used to re-assemble the *E. oligarthrus* genome using SPAdes 3.12 (Bankevich et al., 2012; Safonova et al., 2014). The scaffolds of *E. oligarthus* were obtained with Chromosomer (Tamazian et al., 2016) and using *E. multilocularis* as the reference genome [WormBase ParaSite, Version WBPS12 (WS267)]. Redundant unplaced and unlocalized sequences were discarded from the final assembly. The fragment sequence was considered unplaced if two or more alignments located on different reference sequences or unlocalized if two or more alignments located on the same reference sequences according to Tamazian et al. (2016). The contigs that did not map to the reference genome were included in the final assembly. The standard quality metrics of the assembly such as N50, the total number of contigs and the total length of the assembly were evaluated using QUAST (Gurevich et al., 2013). Putative non-target contigs shorter than 500 bp in length were removed from the final assembly. The completeness of the gene space was validated using BUSCO2 (Simão et al., 2015) and eukaryote CEGGs database. Furthermore, the core of cestodes genes [genes contained in all cestode species according to Maldonado et al. (2017)] was screened using BLAST. Coverage and depth coverage were calculated with custom scripts. Depth coverage refers to the number of times that the same region or position in the reference genome is represented by the assembled genome. Coverage refers to the percentage of the total length of the reference genome that is represented by the assembled genome.

### Gene Prediction and Annotation

The gene annotation of *E. oligarthrus* was performed by transferring the gene annotations from the *E. multilocularis* genome [WormBase ParaSite, Version WBPS12 (WS267)] using a manual and own scripting approach. CDS and protein sequences of *E. multilocularis* and the scaffolds of *E. oligarthrus* were used for this purpose. First, the gene allocated regions were identified using BLAST with an e-value cutoff of 1e−10 and one best target hit. The CDS fragments of *E. oligarthrus* were extracted using bedtools (Quinlan and Hall, 2010; Quinlan, 2014) and the transcripts were re-assembled using Chromosomer (Tamazian et al., 2016). GeneWise (Birney et al., 2004) was used to find the correct frameshift of the CDS and to obtain the final datasets of CDS and proteins. The coordinates of the annotations were assessed using Exonerate (Slater et al., 2005) and added to a final GFF file. The performance of gene annotation and basic statistics for *E. oligarthrus* gene models, including the average intron/exon lengths and the number of introns, were calculated using Eval (Keibler and Brent, 2003). The functional gene annotation was performed using InterProScan-5.7-48.0 (Quevillon et al., 2005) and InterPro2GO databases were used to assign Gene Ontology (GO) terms (Ashburner et al., 2000). Gene models were subjected to BLAST search (Stemmer et al., 2013) against UniprotDB and Blast2GO (https://www.blast2go.com/) was used to define the final annotations and GO terms stats. The GO terms were analyzed using GO.db database implementing R, Bioconductor version: Release (3.8) with custom scripts. Proteins studied in this work were searched using BLAST (Stemmer et al., 2013) against UniProtKB/Swiss-Prot databases. Protein domains were screened against PFAM, and Prosite databases using PFAM_scan (Finn, 2006) or HMMscan 3.0. Global pairwise alignments were performed using Needle software (Rice et al., 2000) in order to identify the orthologous genes related to host–parasite interactions between *E. oligarthrus* and *E. multilocularis* (Brehm and Koziol, 2017). Identity and coverage stats were calculated and only hits with more than 50% of coverage were selected.

### Phylogeny With Nuclear Molecular Markers

Molecular markers of the *Echinococcus* nuclear genome were downloaded from GenBank (Kinkar et al., 2017). For each locus, homologous regions were extracted from complete *Echinococcus* genomes [WormBase ParaSite, Version WBPS12 (WS267)] and the *E. oligarthrus* genome obtained in this work using custom scripts. Homologous genomic sequences from *Taenia solium* were employed as outgroup. The DNA sequences were aligned with ClustalX (v2.0.12) and multiple alignments were edited with BioEdit (v7.1.3). Phylogenetic analyses of concatenated nuclear markers were performed using the maximum likelihood method and Tamura-Nei model implemented by MEGAX software (Kumar et al., 2018). The bootstrap consensus trees inferred from 1000 replicates were retained. The percentage of replicate trees in which the associated taxa clustered together in more than 50% in the bootstrap test are shown for each node. The concatenated data resulted in 1552 bp length for seven taxa. A second phylogeny was calculated using MrBayes 3.1.2 (Ronquist et al., 2012). The evolutionary model was set on generalized time reversible substitution model with gamma-distributed rate variation across the sites and a proportion of invariable sites (GTR +G + I model) with at least 200 samples from the posterior probability distribution, and diagnostics calculated every 1000 generations. We use an exponential prior on the branch length with mean = 0.1 substitutions/site.

### Phylogeny Using Whole-Genome SNPs and Artificial Genome Sequence Construction

Variant calling was performed as described in the “Whole-Genome SNP analysis” section. Genome-wide SNPs were used to perform phylogeny analysis as follows: first, heterozygous SNPs were removed and only the homozygous SNPs with a depth coverage >20× and strictly covered in all the *Echinococcus* species were selected to correct for complete lineage sorting. Afterwards, the homozygous SNP loci were concatenated and the resulting sequence alignment was used to create a phylogenetic tree by implementing the Maximum Likelihood and Bayesian method as above. PartitionFinder 2 (Lanfear et al., 2016) was used to select the best-fit partitioning schemes and models of evolution for the phylogenetic analysis. The selected model was implemented to perform phylogeny analysis.

### Whole-Genome SNP Analysis

Genome sequences of *Echinococcus* species were ordered in chromosomes according to the last version of the *E. multilocularis* genome [WormBase ParaSite, Version WBPS12 (WS267)] using Chromosomer (Tamazian et al., 2016) and were used as mapping templates for the variant calling analyses. Variant calling was performed as follows: first, all of the raw reads from *E. canadensis* G7, *E. granulosus* G1, *E. multilocularis*, and *E. oligarthrus* libraries were processed and filtered by quality. Then, the reads were first mapped against their own reference genomes and then against the genomes of the other three *Echinococcus* species using bowtie2 (Langmead and Salzberg, 2012). Mapping statistics were calculated with Bamtools (Barnett et al., 2011), and duplications were marked and discarded using picard-tools v-2.18 (http://broadinstitute.github.io/picard/). The variant calling was performed using bcftools (Narasimhan et al., 2016; Danecek and McCarthy, 2017) and GATK (McKenna et al., 2010) using the following parameters: variation frequency was set >40% with a depth coverage of at least 70% of the total mean coverage; the base quality of both reference site and variation site was set to >30. Insertion and deletions (indels) were filtered out with VCFtools (Danecek et al., 2011), and SNPs with less than 10 bp far from indels were removed to avoid false-positive SNP. In order to annotate the heterozygous and homozygous polymorphic sites, the reads were first mapped against their own reference genomes and then against the other target genomes. Heterozygous sites were retained only if both forward and reverse reads mapped against the reference and alternative allele at a given nucleotide position whose depth coverage was at least 70% of the total mean coverage supporting that position. Homozygous polymorphic sites were annotated if the forward and reverse reads mapped onto the alternative allele with at least 70% of the total mean coverage supporting that position and if there were no reads supporting the reference allele. Homozygous and heterozygous variant sites were registered for all of the species. The Transition/transversion ratios were calculated using VCFtools (Danecek et al., 2011), and the annotation and classification of SNPs based on the effect of annotated genes were carried out with SnpEff v4.0 (Cingolani et al., 2012). Graphics were built using R software. (https://www.r-project.org/).

## Results

### The *E. oligarthrus* Genome

The *E. oligarthrus* genome was assembled from two paired-end libraries sequenced in Illumina MiSeq. High-quality genomic DNA was purified from two cysts isolated from its natural host, *D. azarae*, which was naturally infected and found in Iguazú National Park, Misiones province, Argentina. Microscopic and macroscopic findings indicated that it was a neotropical *Echinococcus* species. The species and the genotype were confirmed by polymerase chain reaction (PCR) amplification of cytochrome oxidase 1 (cox1) followed by direct sequencing. The *E. oligarthrus* group 1 cox1 sequence was determined (Arrabal et al., 2017). The *E. oligarthrus* genome assembly was performed using a *de novo* assembly strategy, and the best assembly was chosen based on its quality metrics. Due to the type of the metacestode development that consists of close interaction with the host’s tissue and in spite of having done a careful extraction of the parasite material, we sequenced a high proportion of host’s DNA. Indeed, preliminary analysis of raw reads revealed that the sequencing yield of parasite DNA was ∼22%, whereas the remaining ∼78% of the sequences was from presumably *D. azarae*, whose genome has not been sequenced yet. Therefore, we performed a screening of potential contaminants before obtaining the final assembly. Since the genome of *D. azarae* is unknown and non-related sequences are available, we made a thorough identification of sequences truly derived from the target genome using Blob tools software (Laetsch and Blaxter, 2017). This software was specially designed for DNA contamination assessment and is particularly useful for organisms of parasitic origin. Here, we performed a taxonomic selection of the target sequences using the Platyhelminthes taxon (Taxonomy ID: 6157) and created a taxon-annotated GC-coverage plot that allowed us to identify Platyhelminthes contigs and discard all the non-target sequences (**Supplementary Figure 1**). After removing the contaminants, sequences that did match as a top BLAST hit at the phylum level and whose base coverage was >4× and %GC ∼ 41% (typical GC content of *Echinococcus* genus) were used to recover the target reads and re-assemble the *E. oligarthrus* genome (see Materials and methods for more details). The final assembly contained 74513 contigs that were further used to obtain the genome scaffolds. Redundant unplaced and unlocalized sequences (19.7 Mb) were removed from the contigs to obtain the final scaffolds assembly (see Materials and methods for more details). The contigs that did not map to the reference genome (did not allocate in scaffolds) were included in the final assembly. The final *E. oligarthrus* genome assembly was composed of 3764 sequences comprising a total of 86.22 Mb with an average GC content of 41% and showed an N50 ∼ 10 Mb and ∼20× in depth coverage. The assembly metrics for contigs and scaffolds are shown in **Table 1** and **Supplementary Table S1**. The nuclear genome of *E. oligarthrus* showed 76.3% coverage and ∼90% identity with the genome of *E. multilocularis* whose genome size is ∼115 Mb [WormBase ParaSite, Version WBPS12 (WS267)]. The percentage average of nucleotide identity among *Echinococcus* chromosomes ranged from 88.7% to 92.7% (**Supplementary Table S2**). The assembly also included the *E. oligarthrus* mitochondrial genome composed of one scaffold that was obtained from the contigs Eoli_00665 and Eoli_02629. The scaffold length was 13,893 bp with 98% coverage and 96% nucleotide identity to the *E. oligarthrus* mitochondrial reference genome (GenBank accession number AB208545). As expected, the nucleotide identity of the mitochondrial genomes was lower in comparison with other *Echinococcus* species (**Supplementary Figure 2**). To assess the completeness of the genome assembly, we evaluated the gene space using “Benchmarking Universal Single-Copy Orthologs” (BUSCO) (Simão et al., 2015), which measures the genome completeness based on evolutionarily informed expectations of gene content. In this analysis, we identified 77.6% (235 of the 303 core genes) that are expected to be present in all metazoans, including 125 complete and duplicated, 110 fragmented, and 68 missing orthologs. The fragmented nature of the assembly may have prevented many genes from meeting the stringent matching criteria implemented by BUSCO. Indeed, the BLAST results suggest that most of the core genes are identifiable in the genome, even though many genes are present as fragments within the assembly. Also, due to the fragmentation level of the genome assembly, a hybrid and *ab initio* gene prediction is likely impracticable. Since all the available gene annotation transfer tools tried here showed low efficiency, the gene annotation was performed by manual gene annotation transfer as described in the Materials and methods section. First, we used BLAST to search for the best suitable *Echinococcus* species to be used as a template. In this regard, we found that *E. multilocularis* was the most suitable species because of the integrity of the assembly and the higher identity with the genome of *E. oligarthrus* (**Supplementary Table S2**). The final set of *E. oligarthrus* genes comprised 8753 genes coding for proteins (4494 genes with coverage > 50% and 4259 genes with coverage < 50%) (**Supplementary Table S3**). The whole set of genes represented the 85.0% of the total genes expected to be found in species of the genus *Echinococcus*. The predicted proteins were also screened against the conserved core of genes that are present in all the cestodes species according to Maldonado et al. (2017) using BLAST. In this regard, 4872 out of 5203 genes were found for *E. oligarthrus* using e values of <1e−12, which comprised the 93.6% of the total conserved core of genes in cestodes, indicating that the genome contains useful molecular data (**Supplementary Table S4**).

**Table 1:** Genome-wide statistics for the *E. oligarthrus* assembly and gene findings.

Genome statistics	Scaffolds	Contigs
Size of genome (Mb) (*)	86.2	105.9
GC content (%)	41	41
Number of sequences	3764	74,513
N50 (Mb)	10.2	12.83 kb
Gaps (Ns/100 kb)	1366	0
Largest contig (Mb)	16.0	63.7 kb
Deep coverage	20×	20×
Number of predicted genes	8753	
Gene density per Mb	101.5	
Length of proteome (amino acids)	2,009,586	
Maximum protein length (amino acids)	2254	
Average protein length (amino acids)	229	
Average exon length (bp)	203	
Median exon length (bp)	158	
Average exons per transcript	3	
Median exons per transcript	2	
Total length of contained introns (kb)	8622	
Average intron length (bp)	709	
Median intron length (bp)	262	
BUSCO	235/303	162/303

(*) Redundant sequences (19.7 Mb) were removed from the contigs to obtain the final scaffolds assembly.

### *E. oligarthrus* Genes

GO terms were assigned to the 40% of *E. oligarthrus* proteins. In relation to the Molecular Function GO terms frequency, the two main categories found were “binding (GO:0005488)” and “catalytic activity (GO:0003824)”, which is in accordance with the GO terms frequency observed in other cestode genomes (Hahn et al., 2014) and *Echinococcus* species (Maldonado et al., 2017). For the Biological Process GO term, the highest frequencies observed were the categories “cellular process (GO:0009987)” and “metabolic process (GO:0008152)”, also in accordance with the GO terms frequency observed in the related organism (**Supplementary Figure 3**).

From the total gene-set, we searched for genes that have already been described as having a role in host–parasite interactions in *Echinococcus* (Brehm and Koziol, 2017). In this regard, we found 56 genes involved in host–parasite interactions whose coverages and identities to *E. multilocularis* orthologous genes were >50% (**Supplementary Table S5**). Particularly, two genes encoding for the epidermal growth factor (EGF) tyrosine kinase receptors (Eoli_000075800 and Eoli_000617300) with a high percentage of identity (59.8% and 52.6%) and coverage (65.3% and 51.4%) were identified. Also, we found a gene encoding for fibroblast growth factor (FGF) receptor tyrosine kinase (Eoli_000833200) whose percentage of identity and coverage values were 76% and 100%, respectively. Regarding nuclear receptor hormones, we found nine orthologous genes with high identity and coverage (83.8% and 51.8% on average, respectively) including the cestode-specific nuclear hormone receptor Eoli_000937000. Non-kinase receptors were also found, comprising four genes, including Frizzled G protein-coupled receptors (GPCR) (Eoli_000682100) with high identity and coverage (88.6% and 87.6%, respectively). Finally, we identified the amino acid transporters (DAACS family), lipid binding proteins (FABPs), and antigens (AgB and Eg95), specific and conserved proteins in cestodes/*Echinococcus*.

### Comparative Whole Genome Based on SNPs Analysis

**Figure 1:**
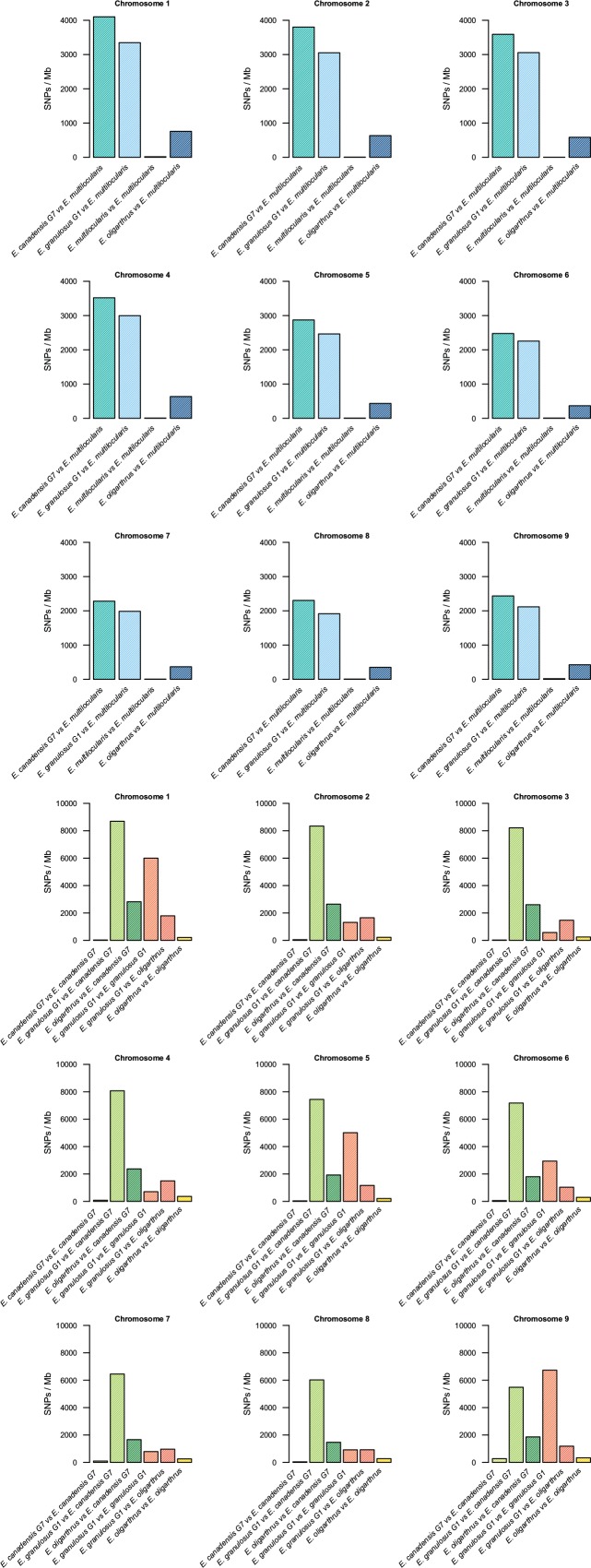
Single-nucleotide polymorphisms in *Echinococcus* chromosomes. Density of single-nucleotide polymorphisms (SNPs/Mb) by chromosome 1–9 among *E. oligarthrus*, *E. multilocularis*, *E. canadensis* G7, and *E. granulosus* G1 species.

The nucleotide variation in the genomes of different *Echinococcus* species was assessed through variant calling analysis. Genetic variants were identified among the genomes of *E. oligarthrus*, *E. multilocularis*, *E. granulosus* G1, and *E. canadensis* G7. Here, we focused on the study of single-nucleotide polymorphisms (SNPs). In order to perform this analysis, NGS raw reads were mapped against a reference genome composed of chromosomes and contigs. For all the analyses, the reads were first mapped against their own reference genomes and then against the genome of the corresponding analyzed species. Homozygous and heterozygous variant sites were identified and were marked in both the reference and the alternative allele (see Materials and Methods for more details). First, we evaluated the intraspecific variation of the *Echinococcus* genomes. As described in Maldonado et al. (2017). *E. granulosus* G1 genome exhibited the highest number of intraspecific variant sites (74,796 SNPs, 0.65 SNPs/kb), followed by *E. oligarthrus* (23,301 SNP, 0.23 SNP/kb), *E. canadensis* G7 (10,791 SNPs, 0.095 SNPs/kb), and *E. multilocularis* (1,287 SNPs, 0.011 SNPs/kb). With regard to the genetic diversity observed among the *Echinococcus* species, we observed that the SNP distribution was similar to the distribution described in our previous research (Maldonado et al., 2017). *E. canadensis* G7 and *E. granulosus* G1 showed a higher number of SNPs (842,322, Ts/Tv = 2.97) between each other than between *E. canadensis* G7 and *E. multilocularis* (314,176, Ts/Tv = 2.97), and in comparison to *E. granulosus* G1 and *E. multilocularis* (272,138, Ts/Tv = 2.94). Furthermore, the pair *E. canadensis* G7 and *E. granulosus* G1 also showed almost 10 orders of magnitude more SNPs than between *E. oligarthrus* and *E. multilocularis* (38,911, Ts/Tv = 3.25). The number of SNPs between *E. oligarthrus* and *E. granulosus* (126,472 Ts/Tv = 2.99) was similar to the number of SNPs between *E. oligarthrus* and *E. canadensis* G7 (171,135, Ts/Tv = 3.00). We also identified homozygous and heterozygous variant sites for the four *Echinococcus* species, for both the reference and the alternative allele in each case. The number of homozygous SNPs was 313,992 for *E. canadensis* G7 and *E. multilocularis*, 266,180 for *E. granulosus* G1 and *E. multilocularis*, 38,762 for *E. oligarthrus* and *E. multilocularis*, 830,768 for *E. canadensis* G7 and *E. granulosus* G1, 125,147 for *E. oligarthrus* and *E. granulosus* G1, and 170,049 for *E. oligarthrus* and *E. canadensis* G7 (**Table 2**). Moreover, the SNP density was assessed in terms of the number of SNPs per 1-Mb length of the *Echinococcus* chromosomes. For all the *Echinococcus* genomes, the highest SNP density (SNPs/Mb of the chromosome) was found for chromosome 1. The chromosomes 2, 3, and 4 showed a slightly lower SNP density than chromosome 1 and chromosomes 5, 6, 7, 8, and 9 showed the lowest SNP density (**Figure 1**). However, the intraspecific SNP density distribution for *E. granulosus* G1 was higher in chromosomes 1, 5, and 9. We also evaluated the SNP distribution of *E. oligarthrus* in the coding and non-coding genomic regions (intergenic, exons, and introns) of *E. multilocularis*. In this regard, the 38,911 SNPs distributed with a higher rate in exons and introns rather than in the intergenic regions. However, those distributed in the coding regions exhibited a higher rate of synonymous changes (67.3%) rather than missense changes (32.6%) (**Supplementary Figure 4**).

**Table 2:** Number and type of SNPs among *Echinococcus* species.

Species	Sample	Alt : Het	Alt : Hom	Total number of SNPs	Ts/Tv
*E. multilocularis*	JAVA05	1152	135	1287	1.98
*E. canadensis* G7	PH14	9937	854	10,791	3.25
*E. granulosus* G1	GH09/3	69,672	5124	74,796	2.32
*E. oligarthrus*	ADA3-ADA5	23,074	227	23,301	2.97
*E. canadensis* G7 vs *E. multilocularis* ^a^	PH14–JAVA05	168	313,992	314,176	2.97
*E. granulosus* G1 vs *E. multilocularis* ^b^	GH09/3–JAVA05	5940	266,180	272,138	2.94
*E. oligarthrus* vs *E. multilocularis* ^c^	ADA–JAVA05	147	38,762	38,911	3.25
*E. canadensis* vs *E. granulosus *G1^d^	PH14–H95/5	1359	830,768	842,322	2.97
*E. oligarthrus* vs *E. granulosus* G1^e^	ADA–H95/5	667	125,147	126,472	2.99
*E. oligarthrus* vs *E. canadensis* G7^f^	ADA3–ADA5–PH14	1044	170,049	171,135	3.00

^a^Ref/Alt : Hom/Hom: 6; Ref/Alt : Hom/Het:0; Ref/Alt : Het/Het:1; Ref/Alt : Het/Hom:9. ^b^Ref/Alt : Hom/Hom: 7; Ref/Alt : Hom/Het:0; Ref/Alt : Het/Het:0; Ref/Alt : Het/Hom:11. ^c^Ref/Alt: Hom/Hom: 0; Ref/Alt : Hom/Het:0; Ref/Alt : Het/Het:0; Ref/Alt : Het/Hom:2. ^d^Ref/Alt : Hom/Hom: 944; Ref/Alt : Hom/Het:0; Ref/Alt : Het/Het:13; Ref/Alt : Het/Hom:9238. ^e^Ref/Alt : Hom/Hom: 55; Ref/Alt : Hom/Het:0; Ref/Alt : Het/Het:0; Ref/Alt : Het/Hom:603. ^f^Ref/Alt : Hom/Hom: 7; Ref/Alt : Hom/Het:0; Ref/Alt : Het/Het:7; Ref/Alt : Het/Hom:35.

### *Echinococcus* Phylogeny Reconstruction by Whole-Genome SNP Analysis

In order to evaluate the contribution of the SNPs to the genetic diversity among the different *Echinococcus* species, we performed phylogenetic analyses by implementing three different approaches: the first used the whole genome variant sites for the four *Echinococcus* species (only the SNPs), which consisted of analyzing 244,246 sites in each genome arising a total of 2.08 Mb; the second approach used genome regions for the four *Echinococcus* species whose depth coverage was >20× and that strictly contained variant sites supported by more than 20 reads, which involved the analysis of 40,179,279 sites in each genome arising in a total of ∼162 Mb. For the third approach, we used only coding regions for the four *Echinococcus* species whose depth coverage was >20× and that strictly contained variant sites supported by more than 20 reads. This involved the analysis of 42,200 sites in each genome arising in a total of 168.8 kb. In all the cases, only the homozygous SNPs were used to perform phylogenetic analyses. The sites and sequences retained here were concatenated and the resulting alignment was used to create the phylogenetic trees by implementing the maximum likelihood and Bayesian methods (see Materials and methods for more details).

**Figure 2:**
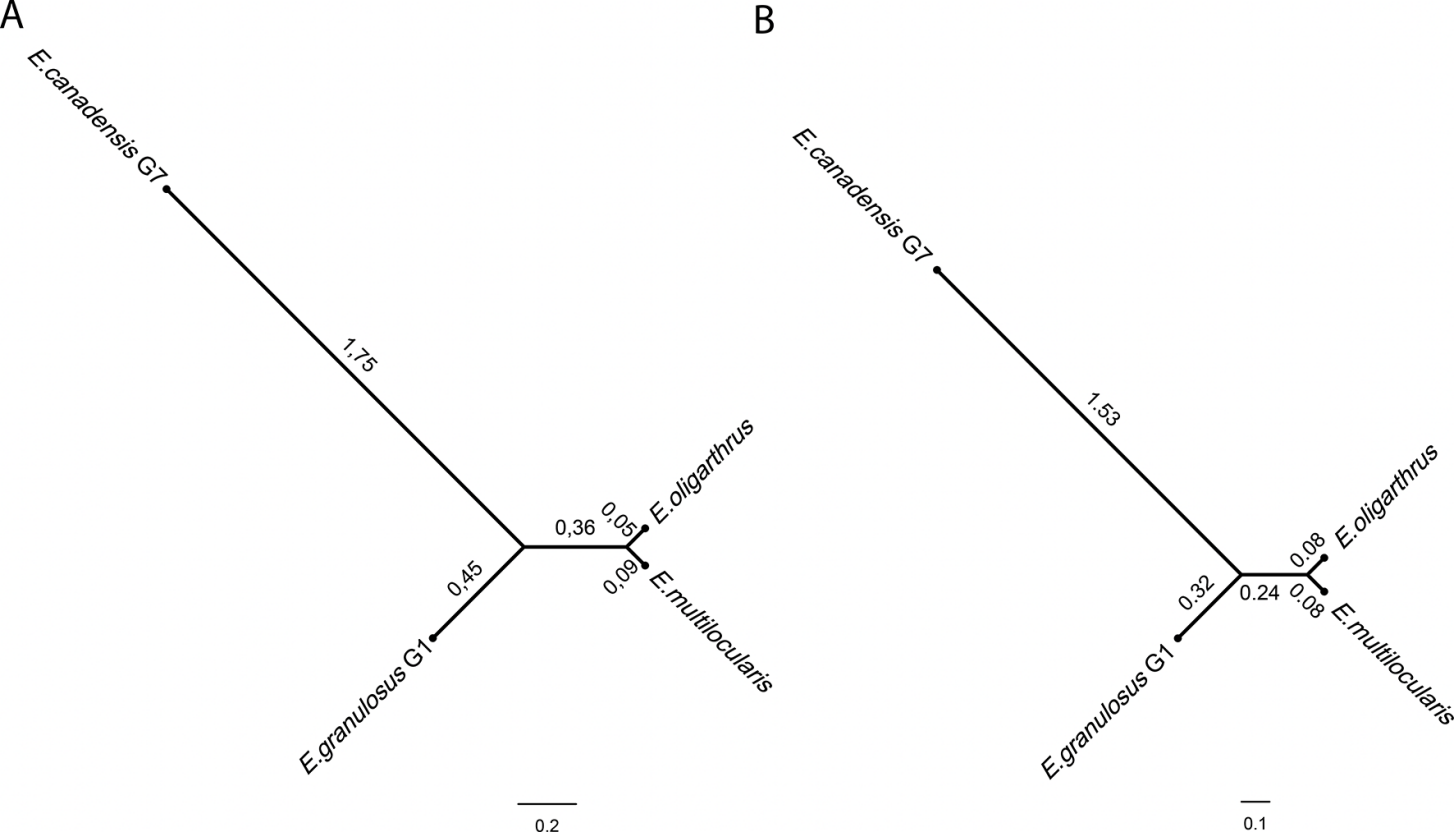
*Echinococcus* phylogenetic tree based on genome-wide nuclear single nucleotide polymorphisms. Phylogenetic tree was constructed using BEAST software using **(A)** GTR + C + I model from four full genome SNPs sequences. **(B)** Transversional substitution model (TVMef) from four full genome SNPs sequences.

The construction of the phylogenetic tree implementing the Bayesian method and using whole-genome SNPs showed a topology that demonstrated a higher genetic distance between *E. canadensis* G7 and *E. granulosus* G1 in comparison with the common node from which *E. multilocularis* and *E. oligarthrus* diverge equidistantly at a very short distance (**Figure 2A**). *E. multilocularis* and *E. oligarthrus* exhibited a low genetic diversity between each other. PartitionFinder 2 (Lanfear et al., 2016) was used to select the best-fit partitioning schemes and models of evolution for the phylogenetic analysis. Transversional substitution model (TVMef) was the best-fitted model. Phylogenetic analysis using TVMef was consistent with the previous result (**Figure 2B**). The topologies of the trees were also consistent with the topology observed using the Maximum Likelihood method (**Supplementary Figure 5A**). Phylogenetic analysis using genomic regions with depth coverage >20× sites and coding regions with depth coverage >20× also showed the same topology, except for the branches that exhibited different lengths (**Supplementary Figures 5B, C**). The total length of the coding sequences used here was ∼42.2 kb and the genes sampled and used for this purpose are listed in **Supplementary Table S6**.

**Figure 3:**
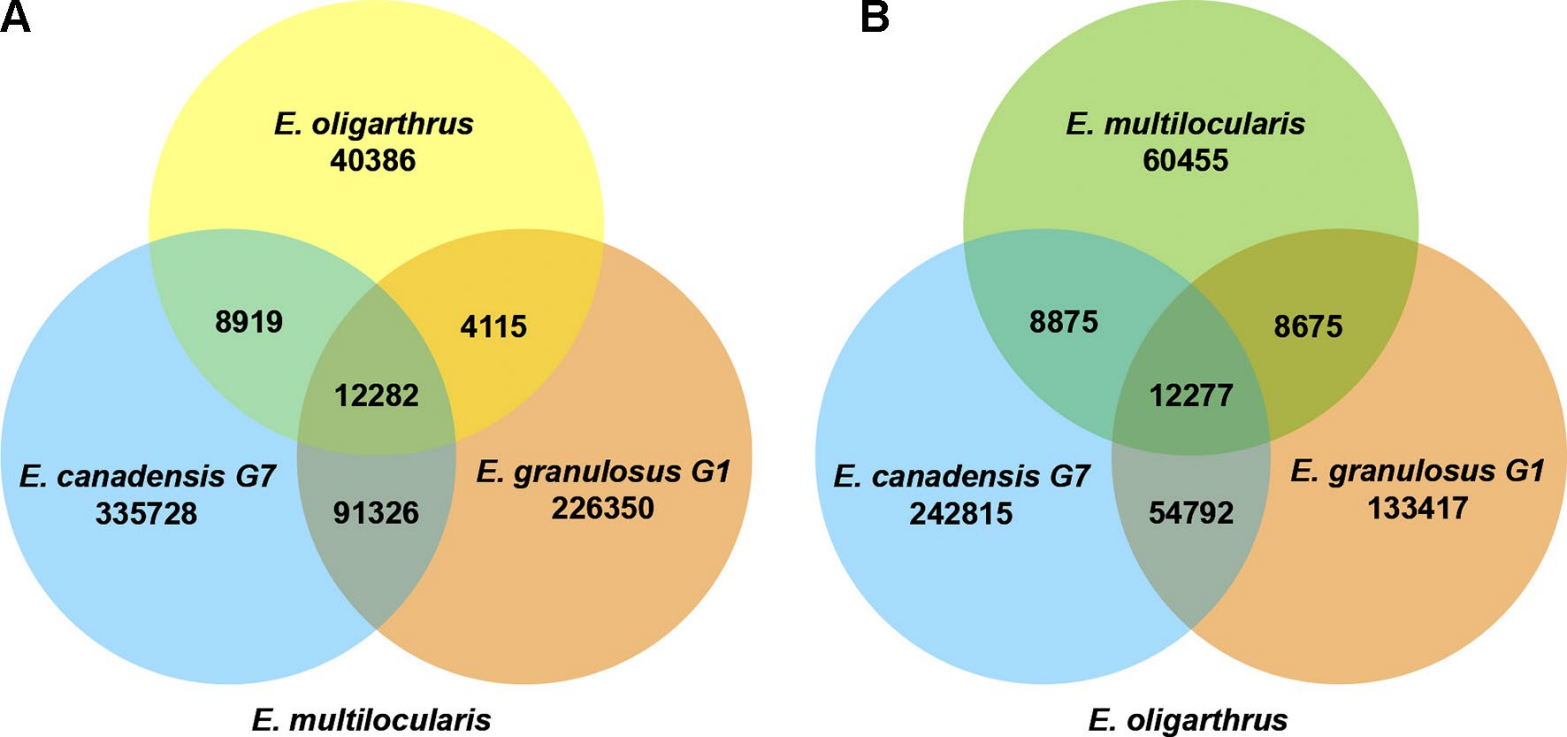
Venn diagram of shared loci between the different *Echinococcus* species using *E. multilocularis* **(A)** and *E. oligarthrus* **(B)** as reference. The numbers under each species name indicate the number of SNPs in the species against the corresponding reference genome. The numbers in the overlap region indicate the number of SNPs with the same polymorphism at the same locus between the species analyzed.

In addition, we registered how many polymorphic loci are shared among the *Echinococcus* species containing the same polymorphism. In previous research, we determined that the number of shared loci was higher when we used *E. multilocularis* as the reference genome, demonstrating to be the basal species and discarding *E.granulosus* G1 and *E. canadensis* G7 as possible candidates (Maldonado et al., 2017). In order to evaluate whether *E. oligarthrus* occupies a basal position in the genus, we incorporated *E. oligarthrus* and analyzed the number of shared loci using both *E. multilocularis* and *E. oligarthrus* as reference genomes and compared the results to each other. Due to the different genome quality assemblies and coverage, we normalized the number of SNPs in shared loci using the effective length of the sampled regions under the assumption that all the genome regions are equally subjected to mutation. In this regard, we found that 12,282 loci shared the same nucleotide change among *E. canadensis* G7, *E. granulosus* G1, and *E. oligarthrus* with respect to *E. multilocularis* (*E. multilocularis* used as reference). Moreover, the number of shared loci between *E. oligarthrus* and *E. granulosus* G1, between *E. oligarthrus* and *E. canadensis* G7, and between *E. canadensis* G7 and *E. granulosus* G1 was 4115, 8919, and 92,326, respectively. Most of the loci were unique for *E. granulosus* G1 (226,350) and *E. canadensis* G7 (335,728) rather than for *E. oligarthrus* (40,386). On the other hand, the number of shared loci containing the same nucleotide change among *E. canadensis* G7, *E. granulosus* G1, and *E. multilocularis* was 12,277 with respect to *E. oligarthrus *(*E. oligarthrus* used as reference). The number of shared loci between *E. multilocularis* and *E. granulosus* G1, between *E. multilocularis* and *E. canadensis* G7, and between *E. canadensis* G7 and *E. granulosus* G1 was 8675, 8875, and 54,792 respectively. Furthermore, and similar to the above results, the highest numbers of unique loci were for *E. granulosus* G1 (133,417) and *E. canadensis* G7 (242,815), rather than for *E. multilocularis* (60,455) (**Figure 3**).

In order to gain accuracy and supporting evidence for our previous results, we reconstructed the *Echinococcus* phylogeny with nuclear molecular markers previously described (Kinkar et al., 2017). Nuclear molecular marker sequences of all the available *Echinococcus* species were concatenated and then were aligned and analyzed using both the Bayesian and maximum likelihood methods. This analysis involved the study of 1552 nucleotides that allowed to root the trees and reinforced the basal position of *E. oligarthrus* (**Supplementary Figure 5D**). The phylogenetic topology obtained was consistent with previous phylogenetic analyses of (Nakao et al., 2013b) that have placed *E. oligarthrus* (and *E. vogeli*) in a basal position for the genus *Echinococcus*. Similar phylogenetic topologies were obtained with the two methods employed here (**Supplementary Figure 5E**).

## Discussion

In this work, we sequenced the first sylvatic species of *Echinococcus*, *E. oligarthrus*, isolated from its natural host and performed comparative genomics between both domestic and sylvatic species. One of the limitations of obtaining complete genomes of wildlife parasites from natural infections resides on the difficulty of obtaining DNA samples free of the host material. In particular, for parasites whose development occurs in intimate contact with the host tissue, such is the case of *E. oligarthrus*. Here, we performed an extensive effort to identify the target sequences of the parasite and assemble the genome. Several steps were applied before the final assembly was obtained, and the final assembly was compared with the *Echinococcus* genomes obtained previously by us (Maldonado et al., 2017). Even though the quality of *E. oligarthrus* genome assembly was lower than other *Echinococcus* genomes, it was high enough to locate the genes and perform comparative genomic analysis.

Hereby, we used these data to unravel the phylogeny of this genus. In previous studies, we described the genetic variation among three *Echinococcus* species (*E. canadensis* G7, *E. granulosus* G1, and *E. multiloccularis*) and assessed the distribution of SNPs in the whole genome as well as the effect and the type of SNPs in the coding regions. In this work, we added a new genome to the analysis of genetic variants and studied the SNP distribution in each one of their chromosomes. Regarding the genetic diversity among the *Echinococcus* species, we found that *E. canadensis* G7 and *E. granulosus* G1 contained almost 10 orders of magnitude more SNPs than between *E. oligarthrus* and *E. multilocularis*. The SNP distribution observed is similar to the distribution described in our previous research (Maldonado et al., 2017) where the genetic diversity within the *E. granulosus sensu lato* species was high. On the other hand, the genetic diversity between the sylvatic species *E. oligarthrus* and *E. multilocularis* is remarkably lower. Furthermore, the genetic variability showed to be unequal for different chromosomes. This fact was revealed by a higher SNP density in larger chromosomes than in the smallest ones. In previous studies, we have also reported a higher gene density in larger chromosomes (Maldonado et al., 2018). However, since most of the SNPs produce synonymous nucleotide changes, the amino acid sequences derived from these genes, even those located in the larger chromosomes, are not altered by the presence of such changes and presumably neither their function.

For several years, the mitochondrial sequences were employed to analyze the *Echinococcus* phylogeny (for a review, see Nakao et al., 2013a). However, the construction of phylogenetic trees based only on mitochondrial DNA data may be biased because it is maternally inherited and, therefore, under particular evolutionary forces that may not represent the evolutionary history for each species (Lymbery, 2017). Indeed, it has been suggested that nuclear sequences should be used when evaluating the phylogenetic positions of new *Echinococcus* isolates (Saarma et al., 2009). Here, we performed maximum likelihood and Bayesian phylogenetic analyses using nuclear DNA sequences and compared the results implementing different models including the best-fitted evolutionary model predicted by PartitionFinder 2 (Lanfear et al., 2016). For this purpose, we used all the shared loci within SNPs that were identified among the *Echinococcus* genomes in both whole genome and coding regions and under the strict criteria of having more than 20× depth coverage in all the species. Hereby, and adding previously described nuclear molecular markers, which provides high accuracy to our results, the tree topology retrieved as the most frequent reconstruction placed *E. oligarthrus* in a basal position. Based on these analyses, we conclude that *E. oligarthrus* may be one of the basal species of the genus *Echinococcus*, together with *E. multilocularis*. These findings also agree with our previous studies (Maldonado et al., 2017) where we proposed a basal sylvatic species that could have accumulated mutations over time until a speciation phenomenon could have given rise to *E. granulosus* G1 and *E. canadensis* G7, which afterwards would have diverged, independently increasing the genetic diversity. The fact that *E. canadensis* G7 and *E. granulosus* G1 share more homozygous polymorphic loci with the same variant supports the hypothesis of a basal sylvatic species. However, since the number of homozygous polymorphic loci with the same variant shared among three of the species is almost equal (12,282 for *E. multilocularis* and 12,277 for *E. oligarthrus* as reference genomes), this result does not allow one to resolve whether *E. multilocularis*, *E. oligarthrus*, or other unknown ancestral related species is the ancestral species from which modern *Echinococcus* genomes could have arisen, and thus remains unclear. This hypothesis could be further probed with the complete genome analyses of more *Echinococcus* species, which would be really useful to describe the complete evolutionary history of these parasites. One of the most interesting implications of the nuclear phylogeny based on SNP analysis found in this work is the position of the *Echinococcus* sylvatic species as basal to the genus *Echinococcus*; in addition, it also demonstrates relevant genetic similarities between *E. multilocuaris* and *E. oligarthrus*. This evolutionary framework may enable data-driven investigation of morphological features and developmental evolutionary studies that would provide relevant information about the neotropical echinococcosis. The generation of genome datasets from additional cestode species would further improve these findings. Phylogenetic studies have allowed the development of hypotheses about the evolutionary history of several taxonomic groups from other perspectives. Such is the case of parasitic organisms and the phylogeography of their hosts that helps to interpret parasite evolution in relation to the migratory patterns of their hosts and vice versa. In the neotropical region, several felids serve as definitive hosts for *E. oligarthrus*. Recently, we determined for the first time the presence of *E. oligarthrus* in ocelot (*Leopardus pardalis*) and the puma (*Puma concolor*) in the north of Argentina using nuclear and mitochondrial molecular markers (Arrabal et al., 2017). Indeed, all the cases reported so far have come from South America (D’Alessandro and Rausch, 2008). In terms of phylogeography, the most suitable explanation is that carnivores originate from immigrants from North America and the ancestral species of *Echinococcus* migrated to South America together with their felid hosts. Early studies suggested that the differentiation of the species of *Leopardus* was likely facilitated by the formation of the Panamanian land bridge. The current hypothesis about felid evolution based on molecular phylogenetic studies suggests that the endemic neotropical felids (genus *Leopardus*) have diverged from other main felid lineages and that before the emergence of the Panamanian isthmus they could have migrated to South America. (Johnson et al., 2006). The finding of an archaic lineage of *Trichinella* in South America also supports the hypothesis of this early carnivore expansion (Pozio et al., 2009). Although originating in North America, the Puma currently has an extensive geographic range in South America and could explain the presence of *E. oligarthrus* in several felid species from the neotropical region. Regarding intermediate hosts, the rodents of the *Hystricomorpha* suborder, natural hosts of *E. oligarthrus*, are known to be the dominant small terrestrial herbivores in South America by the Miocene (Eisenberg et al., 1989). Hence, both the neotropical *Echinococcus* species and their respective hosts seem to have an ancient origin (Nakao et al., 2013b). The need for more genomes and analyses of host–parasite interactions are evident in order to further understand the co-evolution between this parasite and its felid host and the lifestyles of *Echinococcus* species.

Before sequencing the *E. oligarthrus* genome, there was a paucity of related molecular data of this organism. Indeed, only 47 nucleotide sequences have been reported so far, representing only nine genes. Most of them were obtained with the sole purpose of being used as molecular markers (e.g., 18s rRNA nuclear gene or cox1 mitochondrial gene). The scarce sequences information about this parasite was significantly improved through our effort to sequence, assemble, and annotate the genome of *E. oligarthrus*. Despite the fragmented nature of the assembly, we have thoroughly analyzed the gene content in comparisons with other members of the genus. Most of the core genes are identifiable in the genome even though many genes are present as fragments within the assembly. Although many genes presented coverage < 50%, the GO terms distribution found for *E. oligarthrus* was according to that observed in other *Echinococcus* species. Even more, we found that many genes are typically conserved in all the cestodes species. Indeed, these genes composed the core of cestodes genes according to Maldonado et al. (2017), which means that these genes can be found in all the cestodes species whose genomes have been sequenced so far. Therefore, both the genome and the gene annotations of *E. oligarthrus* are suitable to be used in several bioinformatic and comparative analysis as well as to guide hummed and molecular assays. Several works have reported the molecular and cellular mechanisms implicated in the larvae development of *E. multilocularis* and *E. granulosus sensu stricto* (Brehm and Koziol, 2017) but not much is known about the neotropical *Echinococcus* species that have a particular larvae morphology (D’Alessandro and Rausch, 2008). By means of this new genome, we expanded the repertoire of the available genes and reached the 85% of the genes expected to be present in *Echinococcus*, providing for the first time a large set of proteins of a neotropical *Echinococcus* species that can be further studied. Until now, only two families of coding genes implicated in host–parasite interactions had been sequenced from *E. oligarthrus*, the antigen Eg95 and the partial sequence of the antigen B (Haag et al., 2006, Haag et al., 2009). In this regard, we searched for proteins already known to be involved in host–parasite interactions, including genes that are responsible for larval development and signaling pathways in *Echinococcus*. Genes such as Wnt or TNFα receptor and putative regulatory genomic sequences of thousands of important genes were described here. By means of this work, the study of the genes and genomic regulatory regions of the neotropical species *E. oligarthrus* are now reachable by the scientific community. The data obtained here will allow the design of data-driven experiments of gene expression that will provide clues about the particular behavior of the parasite into the mammalian hosts and its differences between sylvatic and domestic species.

## Data Availability

The assembled sequences of the E. oligarthrus genome were deposited in ENA (BioProject PRJEB31222, http://www.ebi.ac.uk/ena/data/view/PRJEB31222). The sequences and the annotation data can also be downloaded from our FlatDB project web page (http://www.bmhid.org/flatdb/). All the data generated and analyzed in this study are included in this published article and within the **Supplementary Material**.

## Author Contributions

LM performed the bioinformatics analysis and wrote the manuscript. JA collected the parasite material and performed the genetic and morphological analysis. LM and LK designed the study. GO constructed the libraries and performed the sequencing. LM, LK, MR, and GO wrote and revised the manuscript. All authors read and approved the manuscript.

## Funding

This study was supported by the MinCyT CAPES BR/RED 1413 (L.K.), Secretaria de Políticas Universitarias-Ministerio de Educación, Cultura, Ciencia y Tecnología, Argentina (CAPG-BA 070/13) (L.K. and G.O.) and Sistema Nacional de Computación de Alto Desempeño (SNCAD-MiNCyT) (L.K.).

## Conflict of Interest Statement

The authors declare that the research was conducted in the absence of any commercial or financial relationships that could be construed as a potential conflict of interest.
