# Supplementary material for: Revisiting the Phylogenetic History of Helminths Through Genomics, the Case of the New Echinococcus oligarthrus Genome
Source: Front Genet. 2019 Aug 7;10:708. doi: 10.3389/fgene.2019.00708 (PMC6692484; doi:10.3389/fgene.2019.00708)
Supplement: Supplementary file 2 [file DataSheet_1.pdf]

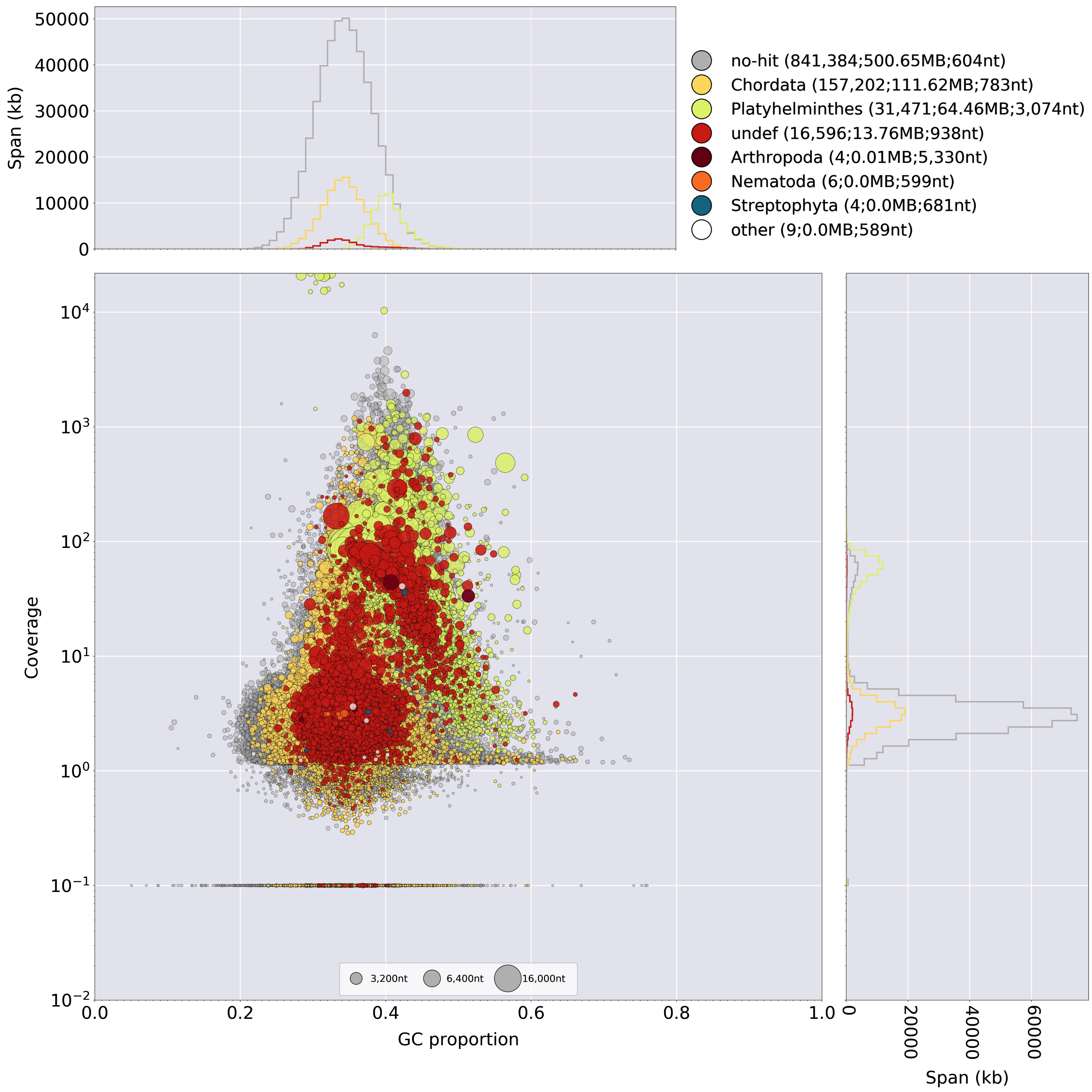

Supplementary figure 1: BlobPlots of preliminary E. oligarthrus assembly: Sequences are represented by circles (with diameters proportional to sequence length) in the scatter plot and coloured by taxonomic affiliation. The order in the legends reflects the taxonomic affiliation of sequences, lists count, total span and N50 by taxonomic group.
