# Supplementary material for: Revisiting the Phylogenetic History of Helminths Through Genomics, the Case of the New Echinococcus oligarthrus Genome
Source: Front Genet. 2019 Aug 7;10:708. doi: 10.3389/fgene.2019.00708 (PMC6692484; doi:10.3389/fgene.2019.00708)
Supplement: Supplementary file 4 [file DataSheet_3.pdf]

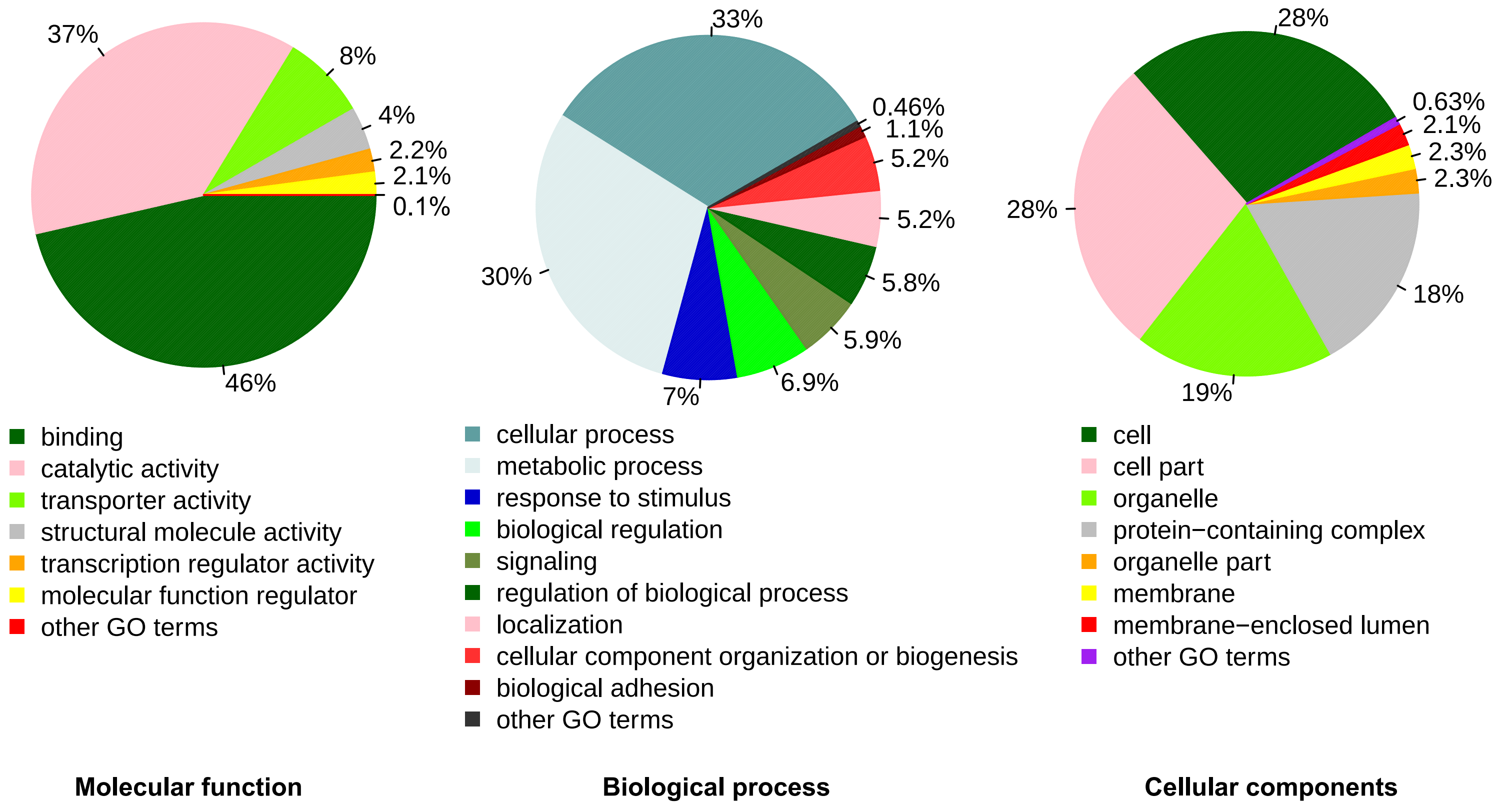

Supplementary figure 3: GO terms main categories of *E. oligarthrus* genes: molecular function, biological process and cellular components.
