# Supplementary material for: Revisiting the Phylogenetic History of Helminths Through Genomics, the Case of the New Echinococcus oligarthrus Genome
Source: Front Genet. 2019 Aug 7;10:708. doi: 10.3389/fgene.2019.00708 (PMC6692484; doi:10.3389/fgene.2019.00708)
Supplement: Supplementary file 5 [file DataSheet_4.pdf]

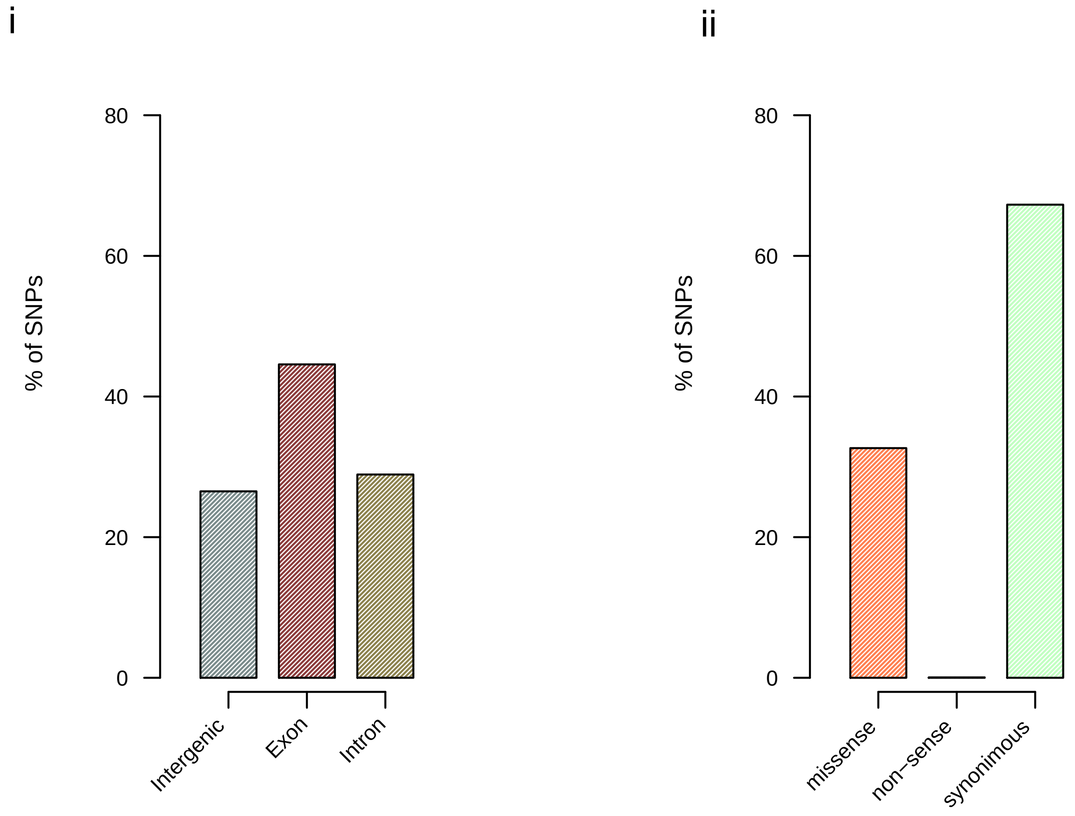

Supplementary figure 4: Single nucleotide polymorphisms in *Echinococcus*. **(i).**Percentage of SNPs distribution in genomic regions defined on the basis of gene architecture between *E. oligarthrus* and *E. multilocularis*. **(ii).**Type of substitution caused by SNPs in coding regions.
