# Supplementary material for: Revisiting the Phylogenetic History of Helminths Through Genomics, the Case of the New Echinococcus oligarthrus Genome
Source: Front Genet. 2019 Aug 7;10:708. doi: 10.3389/fgene.2019.00708 (PMC6692484; doi:10.3389/fgene.2019.00708)
Supplement: Supplementary file 3 [file DataSheet_2.pdf]

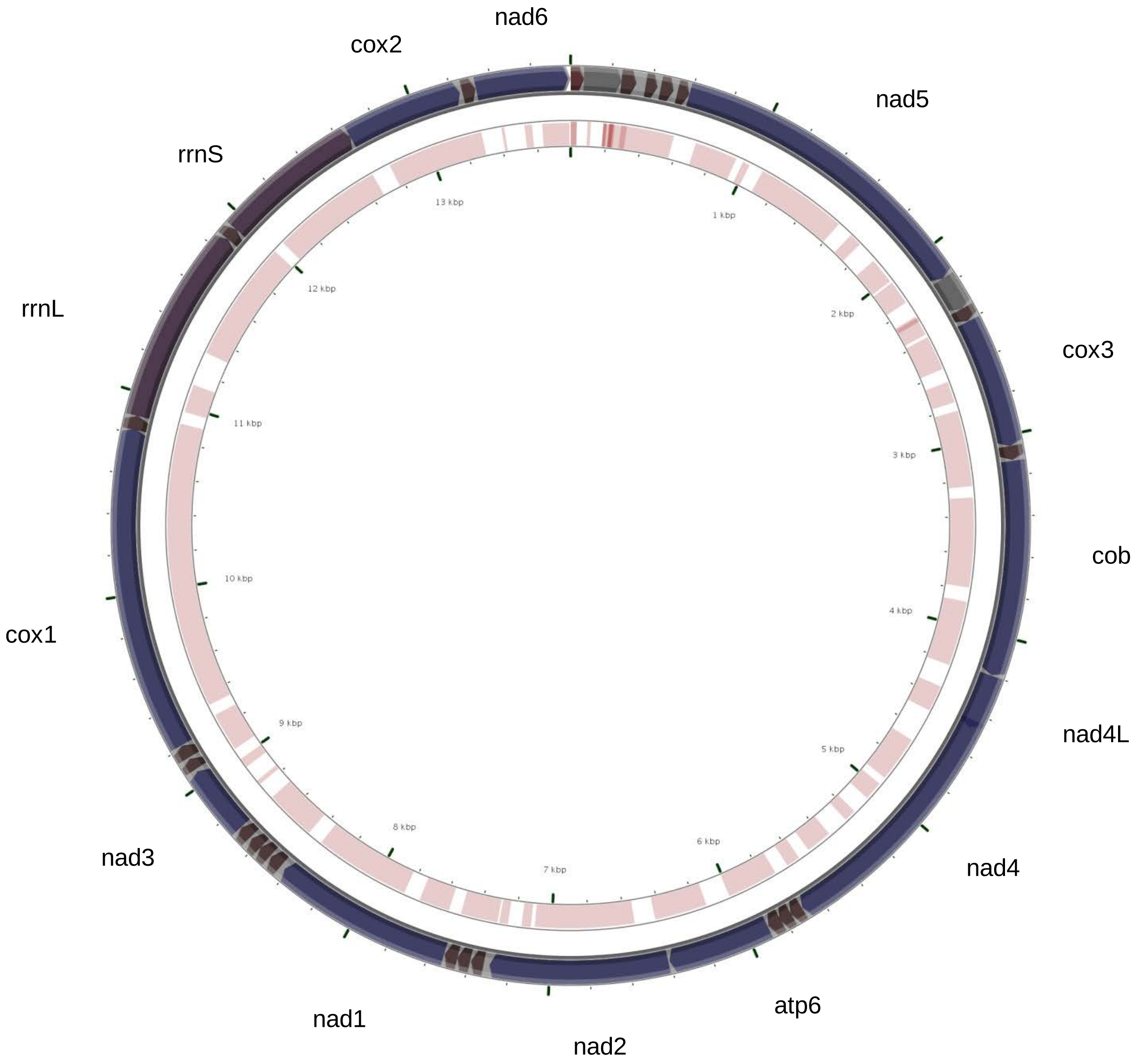

Supplementary Figure 2. *Echinococcus oligarthrus* mitochondrial genome. The mitochondrial scaffold (red circular map) from *E. oligarthrus* was analyzed by sequence identity against reference mitochondrial sequence (GenBak accession number AB208545) (black circular map). All CDS and rRNAs are labelled. tRNAs are shown as arrows.
