# Supplementary material for: Revisiting the Phylogenetic History of Helminths Through Genomics, the Case of the New Echinococcus oligarthrus Genome
Source: Front Genet. 2019 Aug 7;10:708. doi: 10.3389/fgene.2019.00708 (PMC6692484; doi:10.3389/fgene.2019.00708)
Supplement: Supplementary file 6 [file DataSheet_5.pdf]

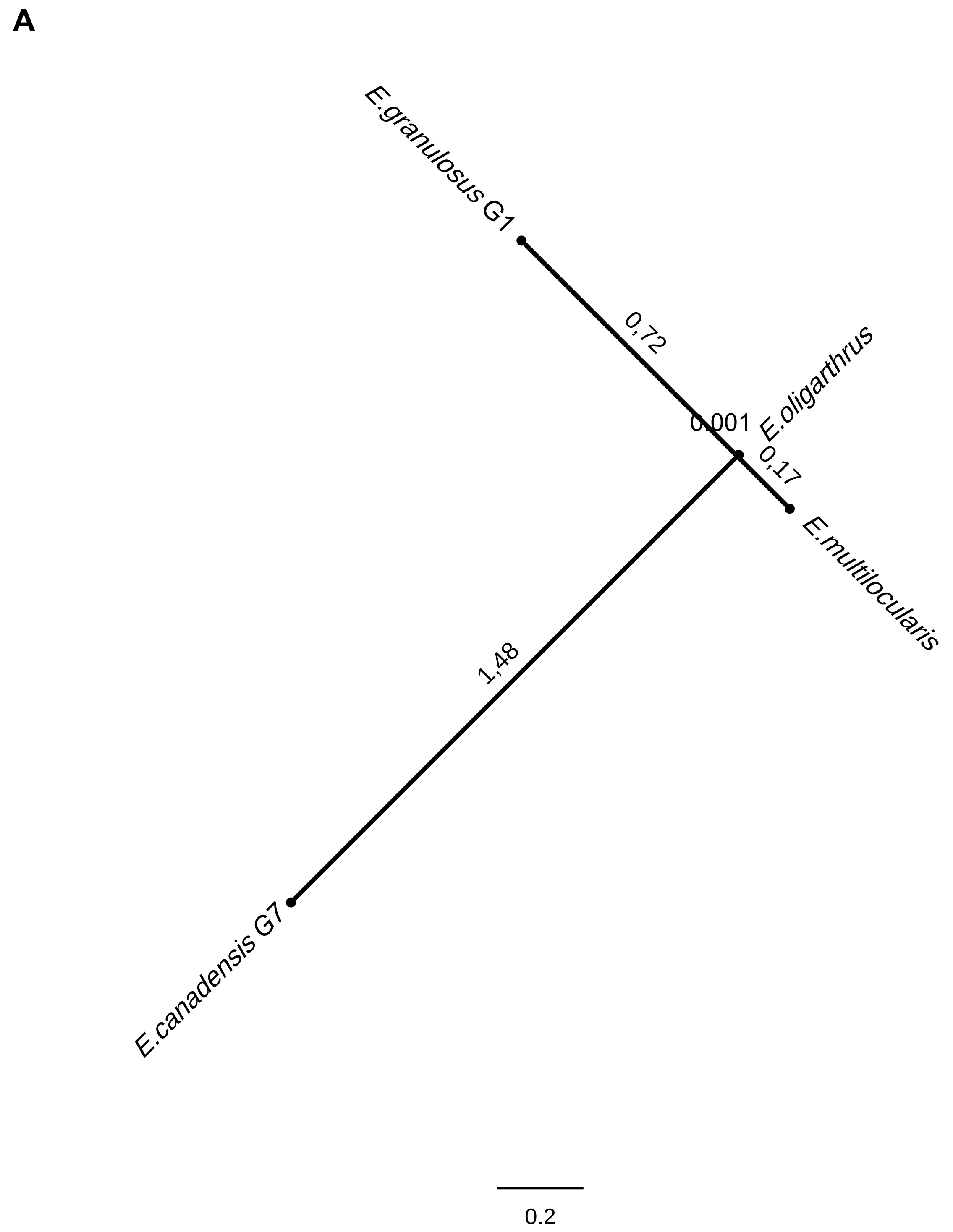
A
E.granulosus G1
0,72
E.oligarthrus
0.001
0,17
E.multilocularis
1,48
E.canadensis G7
0.2

B

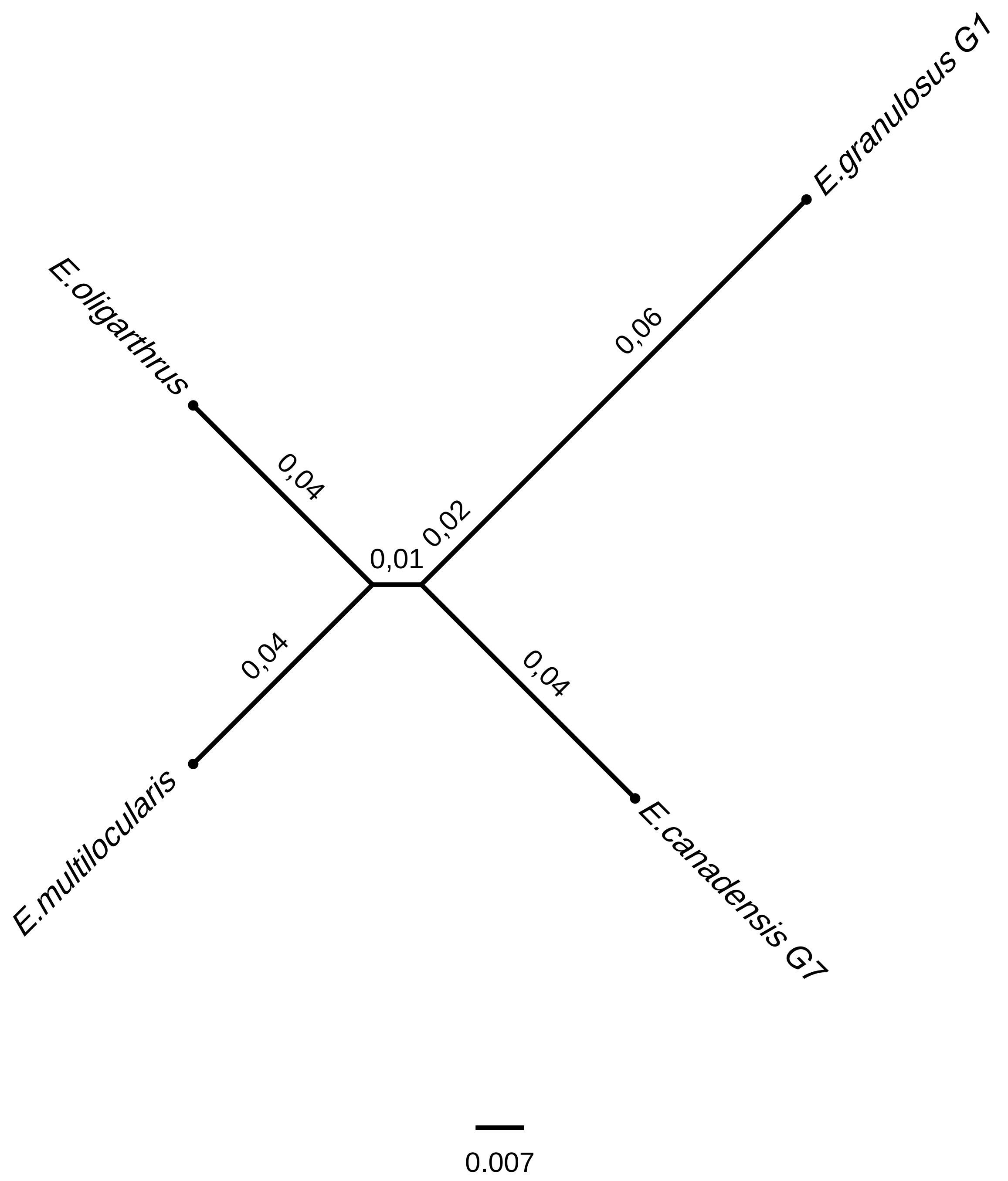
E.granulosus G1
E.oligarthrus
E.multilocularis
E.canadensis G7
0,06
0,04
0,02
0,01
0,04
0,04
0.007

C

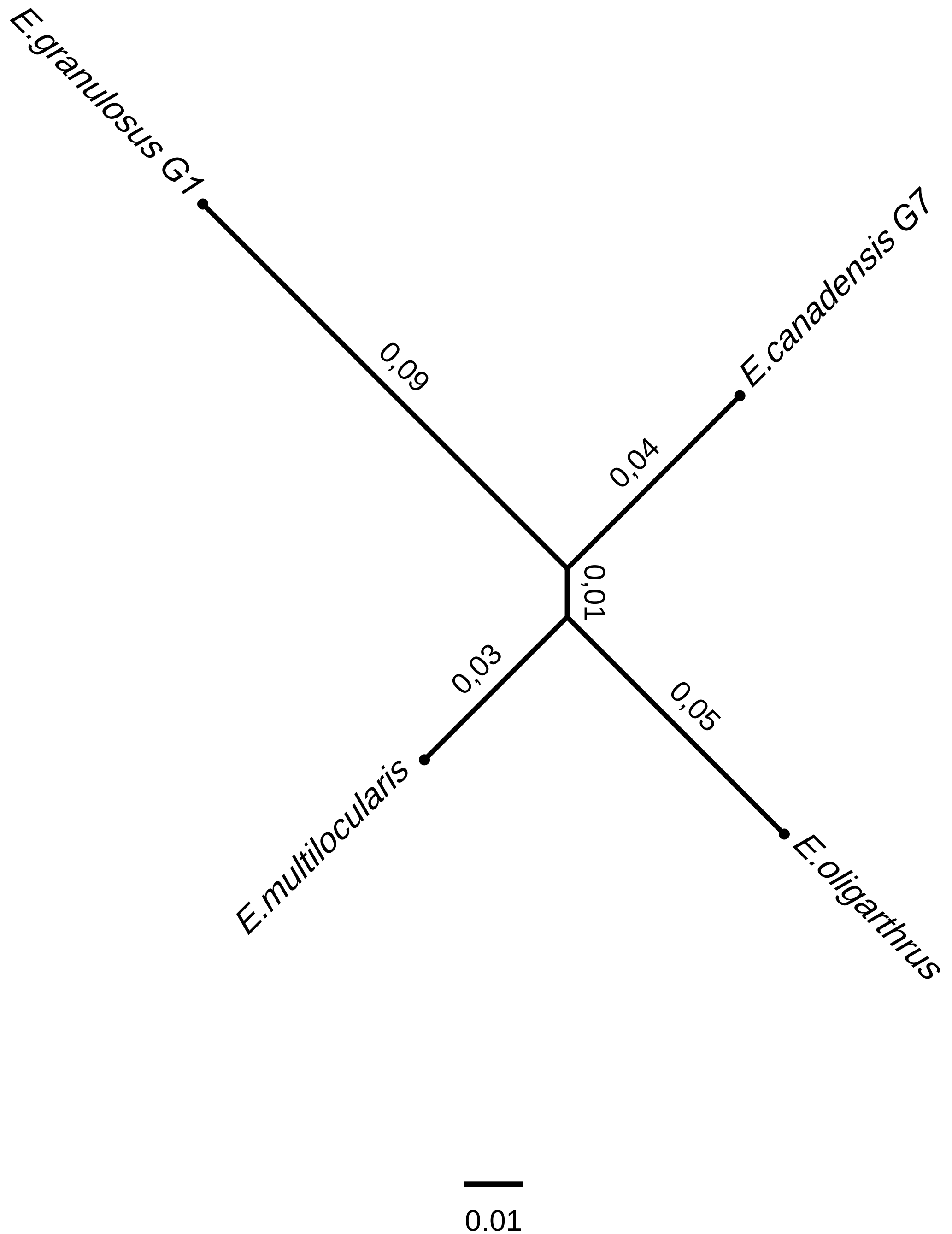
E.granulosus G1
E.canadensis G7
E.multilocularis
E.oligarthrus
0,09
0,04
0,01
0,03
0,05
0.01

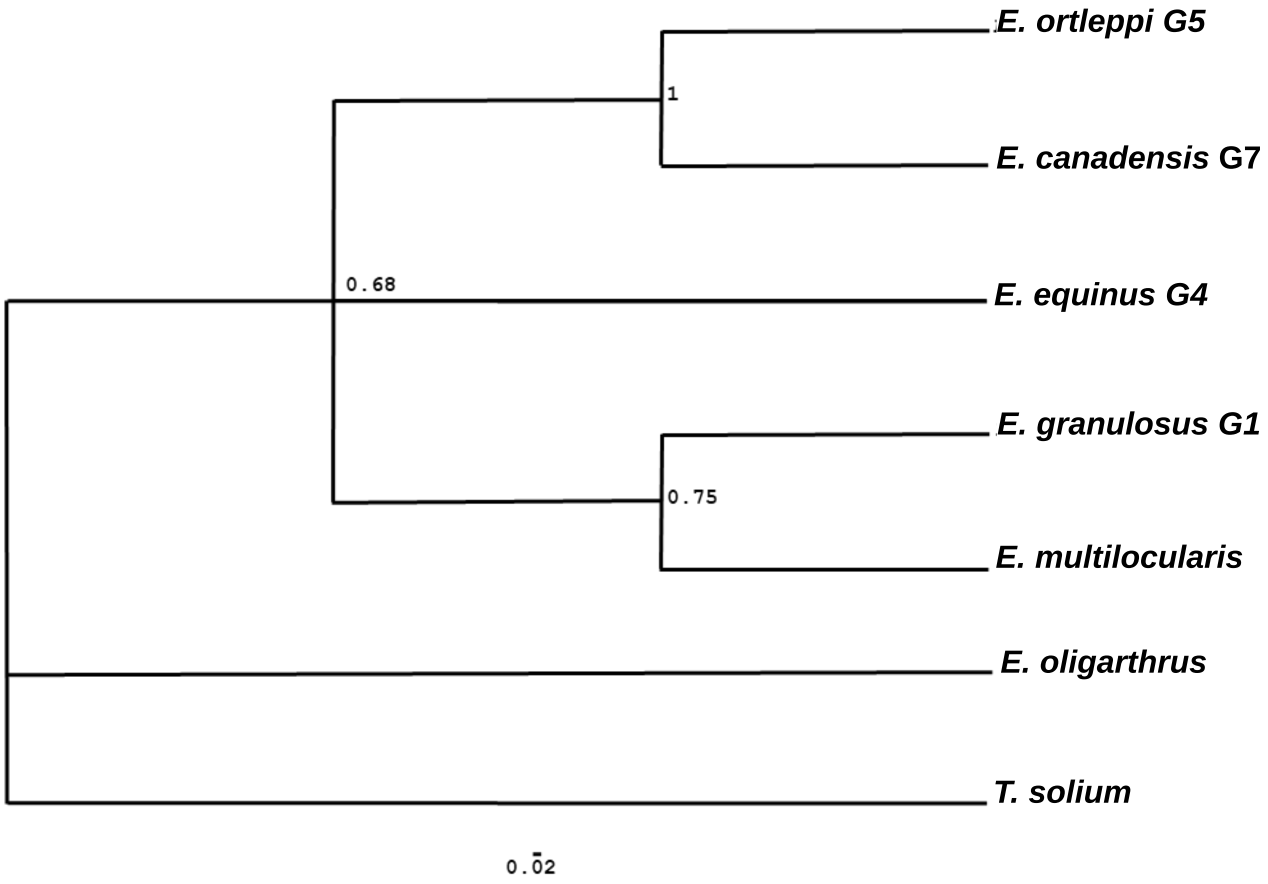

E. ortleppi G5
1
E. canadensis G7
0.68
E. equinus G4
E. granulosus G1
0.75
E. multilocularis
E. oligarthrus
T. solium
0.02

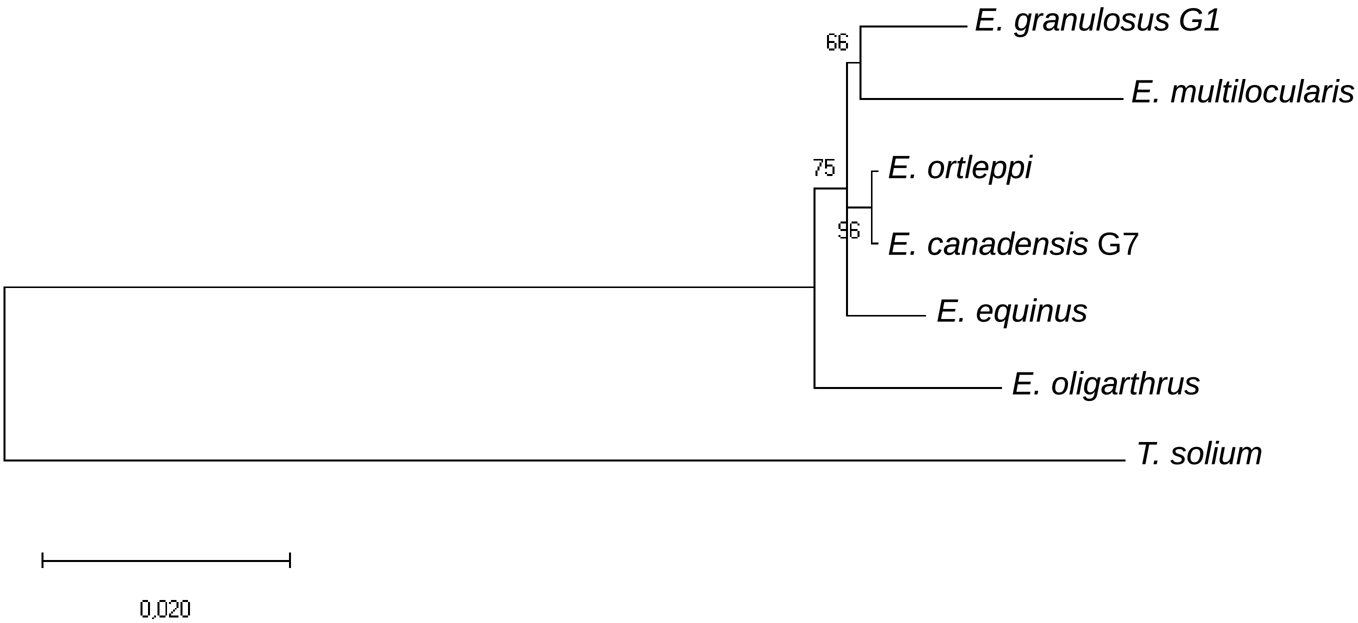

Supplementary figure 5: SNPs and Phylogeny based on genome-wide SNPs analysis. **(A)**.Phylogenetic tree based on genome-wide SNPs analysis by Maximum Likelihood method. **(B)**.Phylogenetic tree based on coding regions SNPs analysis by Bayesian method. **(C).**Phylogenetic tree based on coding regions SNPs analysis by Maximum Likelihood method. **(D).***Echinococcus* phylogeny based on nuclear molecular markers. Phylogenetic tree was obtained by Bayesian analysis. Branch lengths represent the number of substitutions per site. Generalised time reversible substitution model with gamma-distributed rate variation across sites and a proportion of invariable sites (GTR +G + I model) was used. Bayesian posterior probability is shown at each node. A total of 1552 bases were analysed. Taenia. solium species was used as outgroup. **(E).***Echinococcus* phylogenetic tree based on nuclear molecular markers. Phylogenetic tree was obtained by using the Maximum Likelihood method and General Time Reversible model implemented in MEGA X. The percentage of trees in which the associated taxa clustered together is shown next to the branches. Branch lengths represents the number of substitutions per site. A total of 1552bp were analysed. T. solium specie was used as outgroup.
